# A Case of Injury of the Head with Loss of a Portion of the Brain and Recovery

**Published:** 1842-01

**Authors:** Tho. H. Todd

**Affiliations:** Starkville, Mississippi


**Art. IV.**—*A case of injury of the Head with loss of a portion of the Brain and recovery.* By Dr. Tho. H. Todd, of Starkville, Mississippi.

At dusk, on the 3d of August, 1838, I was called to visit a boy belonging to Col. R. S. Lyon, ten miles in the country, who had been thrown from a horse early that evening. My brother, Dr. J. J. Todd, reached the patient about 10 o'clock at night. He fonnd the boy, aged 10 or 12, very stupid and dull, yet he was rational and could be roused to a state of consciousness; he had vomited occasionally and had three or four fits before he saw him. On examining the wound he found the bone extensively fractured and very much depressed. Not having prepared himself with instuments to go into an operation; he sent for me to assist him, and to get the necessary instruments. I did not reach the place before 11 o'clock next day, when we proceeded at once to operate.

The boy had been thrown from a horse and the animal kicked him as he fell. The injury was over the right eye, midway between the superciliary ridge and the edge of the hair, lacerating the soft parts an inch and a half. The hemorrhage was profuse at the time, and we could now discover a considerable quantity of cerebral matter adhering to the adjacent parts. After placing the patient upon a table, we lengthened the wound in the integuments, and made a second incision at right-angles with the former, and dissected up the integuments, until the injury done the bone was fairly exposed. The fracture was extensive, and several portions of bone were found driven through the drura-mater, and penetrating the substance of the brain. We elevated and removed all the detached portions of bone, which left an opening something larger than a half dollar piece of silver; when this was done we removed a teaspoonful, perhaps, of the substance of the brain and several coagula of dark color, which was followed by considerable hemorrhage. After the operation was thus far completed we took one stitch to aid the adhesive strips in keeping the lips of the wound in contact, and applied a compress and the six-tailed bandage to keep the dressing to its proper place. A tickling sensation over the abdomen, with a constant desire to scratch and have it rubbed, attended during the whole time of the operation. We left the boy comfortable, though somewhat exhausted.

5th. Patient has rested well; no excitement. Ordered rhubarb and calcined magnesia to move the bowels.

6th. Has rested well and seems cheerful; some excitement; bled him, and gave a dose of ol. ricini, as the bowels have not been moved since last visit. Directed the lancet to be used if excitement returned; diet to consist of water gruel.

7th. Oil operated well; it was found necessary to repeat the bleeding last night; and still there is too much arterial action; bled him moderately which reduced the pulse and produced quietness.

8th. Rested well; appears cheerful; wound beginning to suppurate; tongue furred. Ordered a small dose of calomel and ipecac.

9th. Medicine operated very well; wound suppurating finely; symptoms good as heretofore.

10th. Patient more restless last night than usual; some excitement; the nurse thought he had a fit this morning; mind still clear; no headache; bled twice during the day, which removed the excitement and rendered the skin soft.

12th. Patient doing well; all the symptoms being favorable.

15th. The general symptoms continue favorable; a fungus cerebri has, however, made its appearance since last date for which a compress has been applied.

20th. The fungus seems to be gradually diminishing in size. From this time the boy's improvement was steady. He is now in fine health and his mind is unimpaired.

December, 1841.

